# rs2231142 (421 C>A, Q141K) Is More Functionally Influential than rs2231137 (34 G>A, V12M) on Anticancer Drug Resistance Mediated by the *ABCG2* Haplotype In Vitro

**DOI:** 10.3390/ijms26157428

**Published:** 2025-08-01

**Authors:** Miho Yamashita, Megumi Tsukamoto, Ritsuko Imai, Himari Muramatsu, Hiroshi Nakagawa

**Affiliations:** 1 Department of Applied Biological Chemistry, Graduate School of Bioscience and Biotechnology, Chubu University, 1200 Matsumoto-cho, Kasugai 487-8501, Japan; 2 Department of Applied Biological Chemistry, College of Bioscience and Biotechnology, Chubu University, 1200 Matsumoto-cho, Kasugai 487-8501, Japan

**Keywords:** ATP-binding cassette (ABC) transporter, ABCG2, BCRP, MXR

## Abstract

The ATP-binding cassette transporter ABCG2 plays a critical role in drug pharmacokinetics and multidrug resistance in cancer therapy. Two common nonsynonymous polymorphisms, rs2231137 (V12M) and rs2231142 (Q141K), are associated with altered ABCG2 function, drug response, and disease susceptibility. However, the functional impact of their haplotype remains poorly understood. In this study, we established Flp-In™-293 cell lines stably expressing ABCG2 (12M/141K) and systematically compared their expression and drug resistance profiles with those of cells expressing ABCG2 (12V/141Q) (WT), ABCG2 (12M/141Q), and ABCG2 (12V/141K). The mRNA of ABCG2 (12M/141K) was expressed at levels comparable to those of the other variants in cells. Cells expressing ABCG2 (12M/141K) exhibited significantly higher resistance to mitoxantrone (10.7-fold) and SN-38 (5.99-fold) than the mock cells. While ABCG2 (12M/141Q) conferred the highest resistance among the tested variants, the *ABCG2 (12M/141K)* haplotype showed a trend toward higher mitoxantrone resistance than the ABCG2 (12V/141Q) (WT) (*p* = 0.066), suggesting a haplotype-specific effect. These findings provide novel insights into haplotype-based modulation of ABCG2 function and its contribution to multidrug resistance, with potential implications for optimizing personalized chemotherapy strategies.

## 1. Introduction

Chemotherapy remains the primary strategy for cancer treatment. However, the efficacy of anticancer drugs varies among patients and is influenced by cancer type. Consequently, it is often challenging to accurately predict therapeutic outcomes and the likelihood of adverse effects. Nevertheless, overcoming cancer cell resistance to chemotherapy remains a critical goal. A growing body of evidence suggests that genetic polymorphisms and mutations significantly contribute to the cancer cell resistance. A deeper understanding of the roles of these genetic variations in both patients and tumors could markedly enhance the success rate of chemotherapy, offering renewed hope in the fight against cancer.

**Figure 1:**
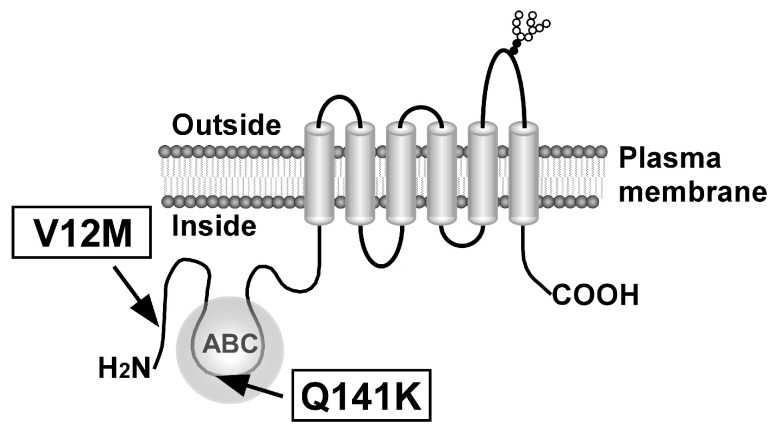
Schematic illustration of human ABCG2 and the locations of SNPs rs2231137 (34 C>A, V12M) and rs2231142 (421 C>A, Q141K). Arrows, locations of SNPs on the ABCG2 protein; ABC, ATP-binding cassette (nucleotide-binding domain).

Several ATP-binding cassette (ABC) transporters, including ABCG2, regulate the intracellular and systemic concentrations of xenobiotics and drugs by actively exporting their substrates across biological membranes. Following the identification of ABCB1 [1,2] and ABCC1 [3], ABCG2 (also known as BCRP/MXR) was discovered in mitoxantrone-resistant human colorectal cancer cells and anthracycline-resistant breast cancer cells [4,5,6], and has since been shown to mediate the efflux of a broad spectrum of anticancer agents [7]. ABCG2 is a 655-amino acid glycoprotein with a molecular weight of approximately 72 kDa, comprising a single ATP-binding domain at the N-terminus and a single transmembrane domain at the C-terminus (Figure 1). The protein functions as a homodimer [8], facilitating the export of a variety of endogenous substrates, including dehydroepiandrosterone sulfate [9], estrone-3-sulfate [9,10], lumichrome [11], porphyrins [12,13,14], and uric acid [15] across the plasma membrane. Overexpression of ABCG2 has been shown to confer resistance to multiple anticancer agents, including camptothecin and its derivatives [7,8,12,16,17,18,19,20], epidermal growth factor receptor tyrosine kinase inhibitors [21,22], mitoxantrone [8,18,19,20,23,24], and methotrexate [25,26]. Moreover, recent clinical investigations have demonstrated that both ABCG2 expression levels and genotypic variants are predictive of disease progression and treatment response in breast carcinoma [27,28], glioma [29], hepatoma [30], lymphoma [31,32], nonpapillary renal cell carcinoma [33], and Non-Small Cell Lung Cancer [34].

Among the single-nucleotide polymorphisms (SNPs) identified in ABCG2, rs2231137 (34 C>A, V12M) and rs2231142 (421 C>A, Q141K) are functionally significant variants that are commonly found in diverse global populations [35]. Imai et al. first reported that the Q141K variant confers reduced drug resistance compared to the wild-type, whereas the V12M variant behaves similarly to the wild-type [18]. Mizuarai et al., on the other hand, showed that both variants confer reduced resistance to ABCG2 substrates compared to the wild-type [19]. Tamura et al. further confirmed reduced drug resistance for the Q141K variant, while reporting increased resistance for the V12M variant [20,36]. Mizuarai et al. also demonstrated that both variants exhibit reduced substrate transport activities [19]. In contrast, Kondo et al. found that the transport activities of the V12M and Q141K variants, when normalized to protein expression levels, were comparable to those of the wild-type [9]. These findings indicate that the functional impact of the V12M and Q141K variants on ABCG2 remains to be fully elucidated. More recently, the *ABCG2 (12M/141K)* haplotype, in which both variants reside on the same allele, has been reported in West Indian individuals [37] and the Han Chinese populations [27,28,38] and has been investigated for its association with susceptibility to antiretroviral therapy–induced hepatotoxicity and gout, respectively. Despite these epidemiological findings, the functional consequences of this haplotype at the cellular level remain unclear.

In the present study, we generated cell lines stably expressing *ABCG2 (12M/141K)* haplotype and systematically compared ABCG2 expression and ABCG2-mediated resistance to anticancer drugs with that of cells expressing *ABCG2 (12V/141Q) (WT)*, *ABCG2 (12M/141Q)*, and *ABCG2 (12V/141K)* haplotypes. Finally, we present novel and significant findings regarding the functional impact of the *ABCG2 (12M/141K)* haplotype. Our results suggested a potential contribution to ABCG2-mediated anticancer drug resistance.

## 2. Results

### 2.1. Levels of ABCG2 mRNA and Protein in Cells Expressing ABCG2 (12M/141K)

**Figure 2:**
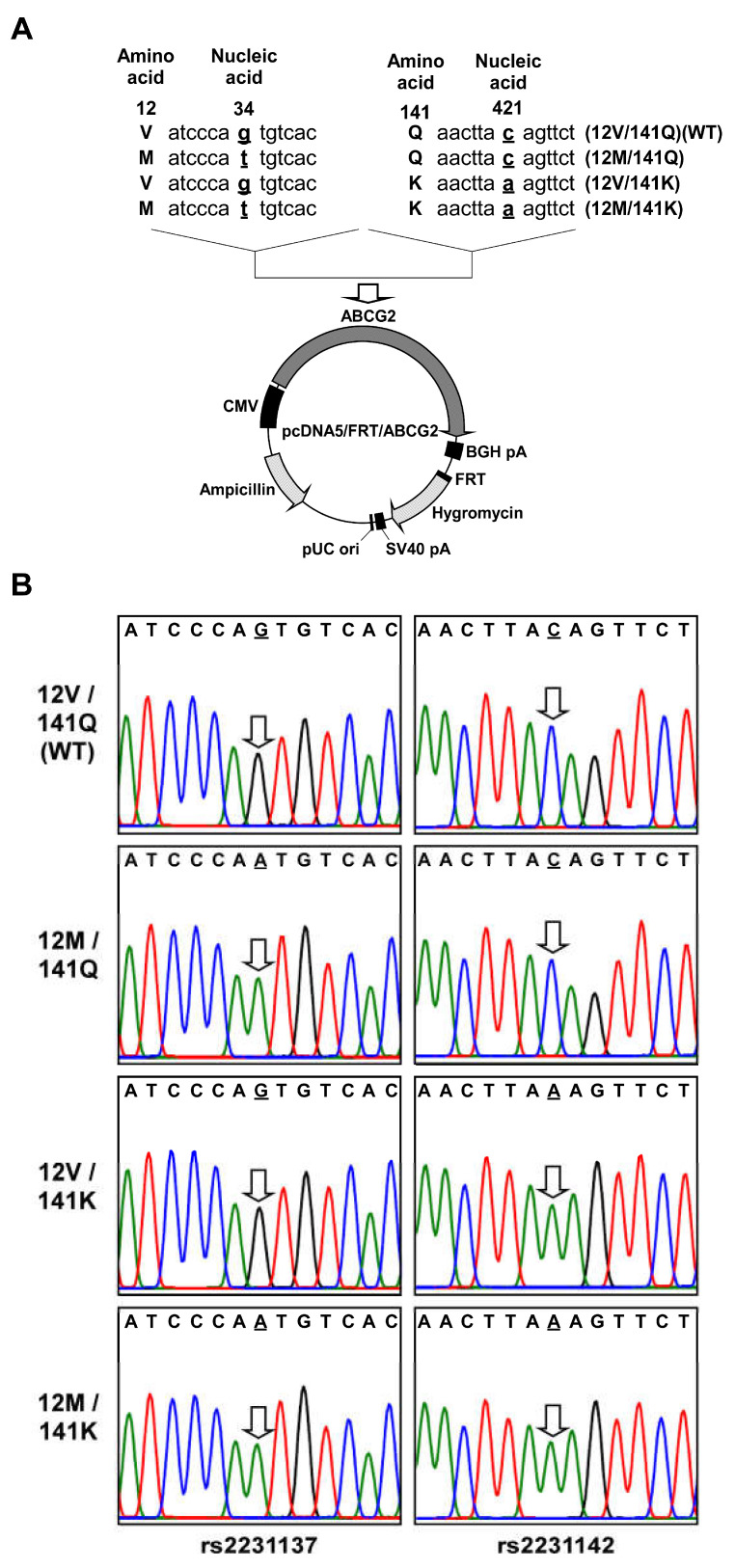
(**A**) Schematic illustration of the pcDNA5/FRT/*ABCG2* expression vector. Partial cDNA sequences of ABCG2 at nucleotide positions 25–45 (V12M) and 412–432 (Q141K) are shown. The variant nucleotides at positions 34 (G>A, V12M) and 421 (C>A, Q141K) are underlined in bold text. ABCG2, ATP-binding cassette subfamily G member 2; BGH, bovine growth hormone; CMV, cytomegalovirus; pA, polyadenylation signal; pUC ori, pUC vector origin of replication; SV40, simian virus 40. (**B**) Electropherograms confirming nucleotide substitutions (C>A) at positions 34 and 421 in the ABCG2 cDNA. Arrows indicate the positions of the substituted nucleotides.

**Figure 3:**
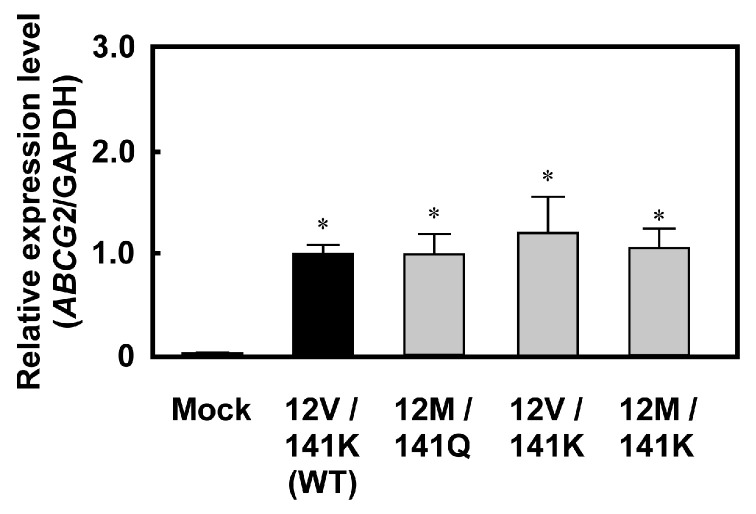
mRNA expression levels of *ABCG2* in Flp-In™-293 cells expressing *ABCG2* haplotypes (*12V/141Q*, *12M/141Q*, *12V/141K*, and *12M/141K*). Expression levels are shown as ratios to GAPDH mRNA and normalized to the ABCG2/GAPDH ratio in WT cells. Data are presented as the mean ± S.D. (*n* = 5). Relative *ABCG2* expression levels were calculated by normalizing to GAPDH and are shown relative to the 12V/141Q group (WT), which was set as 1.0. Statistical analysis was performed using one-way analysis of variance (ANOVA) followed by Tukey’s HSD test (* *p* < 0.01 compared to the mock group).

Stable cell lines expressing ABCG2 (12M/141K) were established using the Flp-In™ system and Flp-In™-293 cells (Figure 1 and Figure 2). Flp-In™-293 cells were transfected with cDNA encoding ABCG2 (12M/141K), which was subsequently integrated into the FRT site of the host genomic DNA. Transfected cells were selected using hygromycin B, and the hygromycin B-resistant clones were further analyzed. To evaluate ABCG2 expression, the mRNA levels of ABCG2 and GAPDH were quantified using qPCR. *ABCG2* mRNA expression in Flp-In-293/ABCG2 (12M/141K) cells was compared to that in Flp-In-293/Mock, Flp-In-293/ABCG2 (12M/141Q), and Flp-In-293/ABCG2 (12V/141K) cells to validate the functionality of the Flp-In™ system in ABCG2-transfected cell lines. Total RNA was extracted from Flp-In-293/Mock, Flp-In-293/ABCG2 (12M/141Q), Flp-In-293/ABCG2 (12V/141K), and Flp-In-293/ABCG2 (12M/141K) cells. RNA quality was confirmed by assessing the A260/A280 ratios and comparing qPCR threshold cycle (Ct) values across all samples. Because the total RNA quality was consistent across groups, *ABCG2* mRNA levels were normalized to GAPDH expression and compared. As shown in Figure 3, *ABCG2* mRNA levels in the ABCG2 cDNA-transfected cells [Flp-In-293/ABCG2 (12V/141Q) (WT), Flp-In-293/ABCG2 (12M/141Q), Flp-In-293/ABCG2 (12V/141K), and Flp-In-293/ABCG2 (12M/141K)] were more than 100-fold higher than those observed in Flp-In-293/Mock cells. Furthermore, *ABCG2* mRNA expression levels were comparable among the Flp-In-293/ABCG2 (12M/141Q), Flp-In-293/ABCG2 (12V/141K), and Flp-In-293/ABCG2 (12M/141K) cell lines, confirming that the Flp-In™ system enabled consistent ABCG2 expression in the established cell lines used in this study.

**Figure 4:**
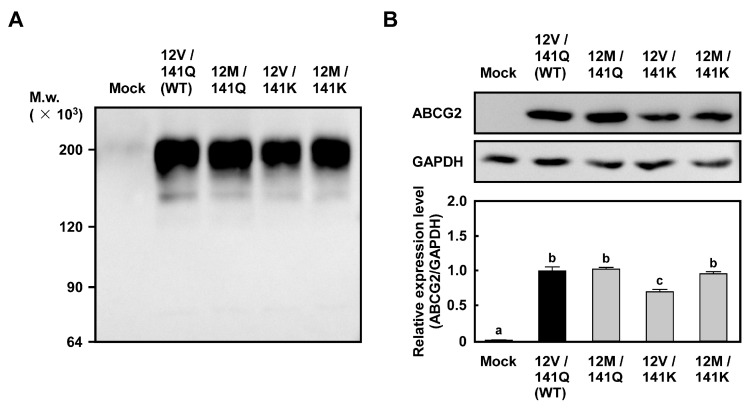
Expression status (**A**) and levels (**B**) of ABCG2 in Flp-In™-293 cells expressing *ABCG2* haplotypes (*12V/141Q*, *12M/141Q*, *12V/141K*, and *12M/141K*). (**A**) Expression status of *ABCG2* haplotypes (*12V/141Q*, *12M/141Q*, *12V/141K*, and *12M/141K*). (**B**) Expression levels of ABCG2 and GAPDH determined by Western blot, as described in the Materials and Methods section. The experiments were independently performed on more than two occasions. Data are presented as the mean ± S.D. (*n* = 3). Statistical analysis was conducted using one-way ANOVA followed by Tukey’s HSD test. Bars with different lowercase letters (a, b, c) indicate statistically significant differences (*p* < 0.05). Relative ABCG2 expression levels were calculated by normalizing to GAPDH and are shown relative to the 12V/141Q group (WT), which was set as 1.0.

To assess the expression status of ABCG2, Western blot analysis was performed under non-reducing conditions. As shown in Figure 4A, ABCG2 (12M/141K) exhibited an electrophoretic mobility nearly identical to that of ABCG2 (12V/141Q) (WT), ABCG2 (12M/141Q), and ABCG2 (12V/141K), suggesting comparable structural characteristics of the glycan moiety among these variants. To quantitatively evaluate ABCG2 expression levels, Western blot was performed using cell lysates treated with PNGase F to remove N-linked glycosylation from ABCG2. As shown in Figure 4B, ABCG2 protein levels in Flp-In-293/ABCG2 (12M/141Q), Flp-In-293/ABCG2 (12V/141K), and Flp-In-293/ABCG2 (12M/141K) cells were significantly higher than those in Flp-In-293/Mock cells. Furthermore, ABCG2 (12M/141K) protein levels were comparable to those of ABCG2 (12V/141Q) (WT) and ABCG2 (12M/141Q), and were significantly higher than those observed for ABCG2 (12V/141K).

### 2.2. Anticancer Drug Resistance of Cells Expressing ABCG2 (12M/141K)

**Figure 5:**
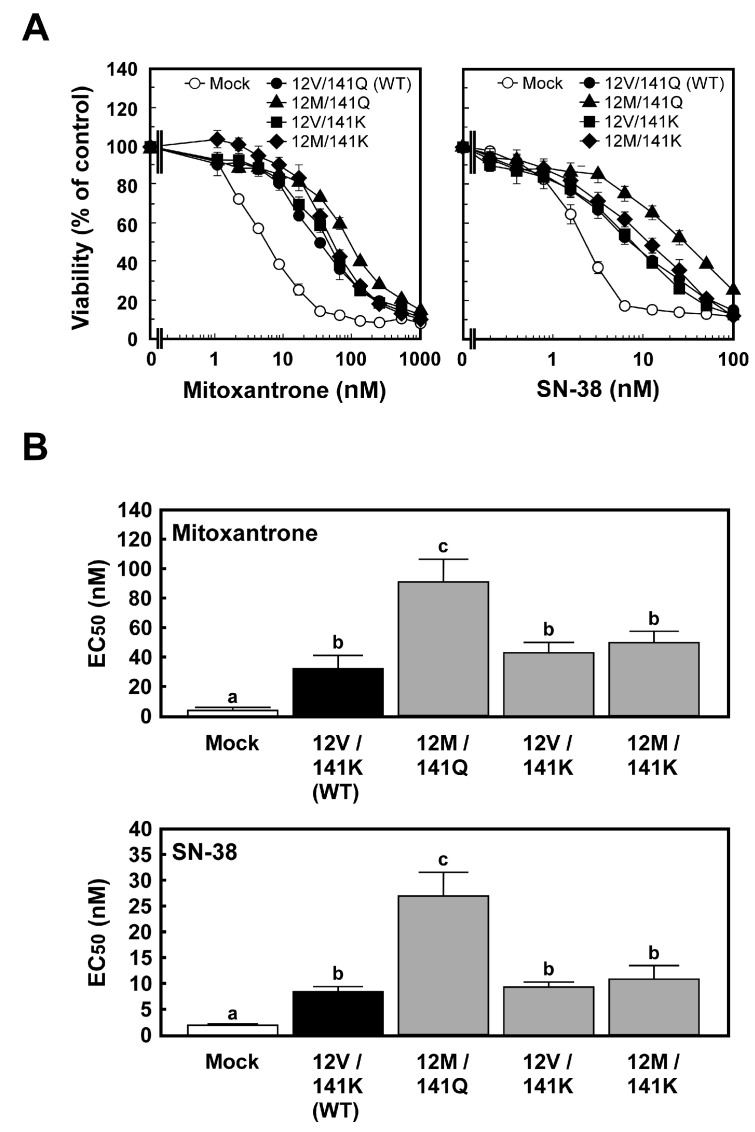
Anticancer drug resistance profiles of Flp-In™-293 cells expressing the *ABCG2* haplotypes (*12V/141Q*, *12M/141Q*, *12V/141K*, and *12M/141K*). (**A**) Representative dose–response curves from five independent experiments are shown. Data are presented as the mean ± S.D. (*n* = 4). (**B**) EC_50_ values calculated from five independent experiments. Data are presented as the mean ± S.D. (*n* = 5). Statistical analysis was performed using one-way analysis of variance (ANOVA), followed by Tukey’s honest significant difference test. Different letters indicate statistically significant differences between the groups (*p* < 0.05).

Having confirmed the consistent expression of ABCG2 variants at both the mRNA and protein levels, we next evaluated whether these variants conferred similar or differential resistance to anticancer drugs. The MTT assay was performed to assess the resistance of Flp-In-293/ABCG2 (12M/141K) cells to anticancer drugs, and comparisons were made with Flp-In-293/Mock, Flp-In-293/ABCG2 (12V/141Q) (WT), Flp-In-293/ABCG2 (12M/141Q), and Flp-In-293/ABCG2 (12V/141K) cells. Flp-In-293/ABCG2 (12V/141Q) (WT) cells expressing wild-type ABCG2 exhibited significantly higher resistance to mitoxantrone and SN-38 than Flp-In-293/Mock cells (Figure 5), which is consistent with the results of previous studies [12,24,36]. As indicated by the EC_50_ values, Flp-In-293/ABCG2 (12V/141Q) (WT) cells exhibited 7.04-fold and 4.42-fold increases in resistance to mitoxantrone and SN-38, respectively, compared to Flp-In-293/Mock cells (Figure 5B and Table 1). Similarly, Flp-In-293/ABCG2 (12M/141K) cells exhibited significantly higher resistance to mitoxantrone and SN-38 compared to Flp-In-293/Mock cells (Figure 5). Based on the EC_50_ values, Flp-In-293/ABCG2 (12M/141K) cells exhibited 10.7-fold and 5.99-fold increases in resistance to mitoxantrone and SN-38, respectively, compared to Flp-In-293/Mock cells (Figure 5B and Table 1). Among the four ABCG2-expressing cell lines, Flp-In-293/ABCG2 (12M/141Q) cells exhibited significantly higher resistance to both mitoxantrone and SN-38 than the other variants (Figure 5 and Table 1). Although no statistically significant differences in EC_50_ values were observed among Flp-In-293/ABCG2 (12V/141Q) (WT), Flp-In-293/ABCG2 (12V/141K), and Flp-In-293/ABCG2 (12M/141K) cells, Flp-In-293/ABCG2 (12M/141K) cells showed a trend toward higher resistance to mitoxantrone than Flp-In-293/ABCG2 (12V/141Q) (WT) cells (*p* = 0.066), suggesting a specific effect of the *ABCG2 (12M/141K)* haplotype.

These findings prompted us to further consider the functional implications of ABCG2 (12M/141K) expression in relation to anticancer drug resistance in the context of known effects of the individual V12M and Q141K variants.

## 3. Discussion

### 3.1. Establishment of Human ABCG2 (12M/141K)-Expressing Cells Using the Flp-In™ System

To quantitatively evaluate the functional impact of the *ABCG2 (12M/141K)* haplotype on drug resistance, Flp-In™-293 cells expressing this variant were established using a site-specific integration system. The drug resistance of cancer cells and the variation in resistance levels among individuals are often attributed to the overexpression of ABC transporters and the presence of single-nucleotide polymorphisms (SNPs) in their genes, as supported by findings from various studies [12,24,36,39]. Our previous research showed that specific SNPs within the ABCG2 gene, such as rs2231137 (V12M), rs2231142 (Q141K), rs1061018 (F208S), rs3116448 (S248P), rs1354553769 (S441N), and rs192169063 (F489L), can affect drug resistance levels in cells expressing ABCG2 [36]. Among these, rs2231137 (V12M) and rs2231142 (Q141K) are two common and functionally important variants associated with disease susceptibility and drug response across diverse pathological contexts, including gout [38,40], cancer [27,29,30,31,32,33,34], chronic kidney disease [41], idiopathic male infertility [42], preeclampsia [43], and type 2 diabetes [44]. However, the combined functional impact of these two SNPs, when present on the same haplotype *(12M/141K)*, has not been elucidated. In this study, we successfully established Flp-In™-293 cells expressing the *ABCG2 (12M/141K)* haplotype to address this question.

In the system employed in this study, a single copy of cDNA was inserted at a specific location (FRT site) in the telomere region of chromosome 12 in Flp-In™-293 cells [36,45,46], allowing quantitative analysis of the effects of SNPs and their combinations on drug resistance. Successful generation of *ABCG2 (12M/141K)* cDNA is shown in Figure 2. Although we did not directly determine the integration site or copy number of *ABCG2 (12M/141K)* cDNA, the mRNA expression levels were comparable to those observed in Flp-In-293/ABCG2 (12V/141Q) (WT), Flp-In-293/ABCG2 (12M/141Q), and Flp-In-293/ABCG2 (12V/141K) cells (Figure 3), strongly suggesting that transfected *ABCG2 (12M/141K)* cDNA was properly integrated into the FRT site. Thus, the copy number of the integrated *ABCG2* cDNA in Flp-In-293/ABCG2 (12M/141K) cells was presumed to be identical to that in Flp-In-293/ABCG2 (12V/141Q) (WT) and Flp-In-293/ABCG2 (12V/141K) cells. This confirmed that the established system is suitable for analyzing the functional impact of the *ABCG2 (12M/141K)* haplotype on drug resistance in vitro.

### 3.2. Anticancer Drug Resistance of Flp-In-293/ABCG2 (12M/141K) Cells

We next examined whether the expression of the *ABCG2 (12M/141K)* haplotype altered cellular resistance to ABCG2 substrate anticancer drugs. ABCG2 has been reported to facilitate the transport of a wide range of xenobiotics (camptothecin and its analogs [7,8,12,16], epidermal growth factor receptor tyrosine kinase inhibitors [21,22], mitoxantrone [8,23,24], methotrexate [25,26]) and certain endobiotics (dehydroepiandrosterone sulfate [9], estrone-3-sulfate [9,10], lumichrome [11], porphyrins [12,13,14], and uric acid [15]). Consistent with our findings and previous studies [7,12,16,23,24,36], Flp-In-293/ABCG2 (12V/141Q) (WT) cells exhibited resistance to the ABCG2 substrate anticancer drugs mitoxantrone and SN-38 compared to Flp-In-293/Mock cells. Flp-In-293/ABCG2 (12M/141K) cells exhibited significantly higher resistance to mitoxantrone and SN-38 than Flp-In-293/Mock cells (Figure 5 and Table 1), demonstrating that ABCG2 (12M/141K) is a functionally active transporter.

In this study, only two representative ABCG2 substrates, mitoxantrone and SN-38, were examined. Future investigations with other ABCG2 substrates are expected to enhance our understanding of the haplotype-based modulation of ABCG2 function and its contribution to multidrug resistance, with potential implications for optimizing personalized chemotherapy strategies. Such substrates include such as dehydroepiandrosterone sulfate [9], estrone-3-sulfate [9,10], lumichrome [11], porphyrins [12,13,14], and uric acid [15], as well as anticancer agents, including camptothecin and its derivatives [7,8,12,16,17], epidermal growth factor receptor tyrosine kinase inhibitors [21,22], mitoxantrone [8,23,24], and methotrexate [25,26].

### 3.3. Comparison of Haplotype-Specific Effects of ABCG2 (12M/141Q) and ABCG2 (12V/141K) on Cellular Resistance to Anticancer Drugs

The single-nucleotide polymorphisms rs2231137 (34 C>A, V12M) and rs2231142 (421 C>A, Q141K) are functionally significant variants that are commonly found in diverse global populations [35]. However, the precise functional impact of the V12M (referred to as ABCG2 [12M/141Q] in the present study) and Q141K (referred to as ABCG2 [12V/141K]) variants on ABCG2 activity remains to be fully elucidated.

Consistent with previous findings by Imai et al. [18] and Tamura et al. [20,36], ABCG2 (12M/141Q)—previously referred to as ABCG2 (V12M)—conferred significantly higher resistance to both mitoxantrone and SN-38 compared to ABCG2 (12V/141Q) (wild-type). In contrast, this finding differs from the report by Mizutani et al. [19], which showed reduced resistance conferred by the V12M variant. Meanwhile, ABCG2 (12V/141K)—referred to as ABCG2 (Q141K) in prior studies—exhibited comparable resistance to mitoxantrone and SN-38 as ABCG2 (12V/141Q) (WT) in our study. This observation differs from previous reports by Imai et al. [18], Mizutani et al. [19], and Tamura et al. [20,36], which showed that the Q141K variant conferred lower resistance to these substrates. These discrepancies may be attributed, at least in part, to differences in the gene expression systems used across studies. Specifically, Imai et al. [18] employed the pcDNA3.1 vector, and Mizuarai et al. [19] used a retroviral vector to express ABCG2. In these systems, although it may be possible to select cell clones with comparable expression levels, it is extremely difficult to ensure identical chromosomal insertion sites. If the transgene is integrated into a transcriptionally active or regulatory region, it may indirectly influence cellular drug resistance and confound functional interpretation. In contrast, our study utilized the Flp-In™ system, which allows for site-specific integration of a single copy of cDNA into a predetermined genomic locus, thereby minimizing variations in the number of inserted gene copies and the surrounding genomic context. Given this methodological advantage, it can be generally considered that, at least theoretically, our results more accurately reflect the true functional impact of the V12M and Q141K variants under controlled expression conditions. One possible explanation for the discrepancy with the findings of Tamura et al. [20,36], who also employed the Flp-In™ system, may lie in the difference in cell seeding density: 2000 cells per well were used in their study, whereas 5000 cells per well were used in the present experiments. Such differences could influence drug exposure and cellular response, thereby affecting the observed resistance levels.

### 3.4. Haplotype-Specific Effects of ABCG2 (12M/141K) on Cellular Resistance to Anticancer Drugs

In this study, we generated Flp-In-293/ABCG2 (12M/141K) cells expressing single-nucleotide polymorphism (SNP) variants rs2231137 (V12M) and rs2231142 (Q141K) of human ABCG2 using the Flp-In™ system. Unlike conventional transfection methods that use the pcDNA3.1 vector or a retroviral vector [18,19], our system allows for controlled copy numbers and specific integration sites of cDNA into the chromosome, as previously described [36]. Flp-In-293/ABCG2 (12M/141K) cells exhibited significantly lower resistance to both mitoxantrone and SN-38 compared to Flp-In-293/ABCG2 (12M/141Q) cells. Since the overall expression levels of ABCG2 (12M/141K) were comparable to those of ABCG2 (12M/141Q), this reduction in drug resistance may be attributed to differences in the amount of ABCG2 localized to the plasma membrane or to altered affinities for mitoxantrone and SN-38. Although no statistically significant differences in EC_50_ values were observed among Flp-In-293/ABCG2 (12V/141Q) (WT), Flp-In-293/ABCG2 (12V/141K), and Flp-In-293/ABCG2 (12M/141K) cells, Flp-In-293/ABCG2 (12M/141K) cells showed a trend toward higher resistance to mitoxantrone than Flp-In-293/ABCG2 (12V/141Q) (WT) cells (*p* = 0.066), suggesting a haplotype-specific effect of the ABCG2 (12M/141K).

Previous studies have shown that drug resistance profiles can be affected by the expression levels of ABC transporters [47,48]. Previous studies have shown that nonsynonymous SNPs within the *ABCG2* gene can affect the function of the transporter by altering substrate specificity, intracellular localization, and protein stability, even when expression levels are unchanged [12,39]. In this study, the expression levels of ABCG2 (12M/141K) in Flp-In-293/ABCG2 (12M/141K) cells were comparable to those in Flp-In-293/ABCG2 (12M/141Q) cells, yet ABCG2 (12M/141K) cells showed lower resistance to mitoxantrone and SN-38 than Flp-In-293/ABCG2 (12M/141Q) cells. These findings suggest that the combined presence of the V12M and Q141K polymorphisms may increase the intracellular accumulation of mitoxantrone and SN-38 compared to the presence of the V12M polymorphism alone.

### 3.5. Future Perspectives

Our study quantitatively compared the drug resistance conferred by each of the four *ABCG2* haplotypes (*12V/141Q*, *12M/141Q*, *12V/141K*, and *12M/141K*) and demonstrated distinct differences in their drug resistance profiles. However, further investigations are required at both the cellular and molecular levels to gain a more precise understanding of the molecular mechanisms underlying these findings. At the cellular level, analyses of the intracellular distribution of ABCG2 (12M/141K) by using 5D3 antibody [39] and the intracellular accumulation of test drugs by using HPLC or LC-MS/MS will be important to clarify the impact of these haplotypes based on the intracellular behavior of ABCG2, in addition to its protein expression levels (Figure 4B). At the molecular level, evaluating the transport activity of ABCG2 (12M/141K) by using membrane vesicles, followed by molecular docking simulations with AutoDock, is expected to help gain a deeper understanding of how the Val12-to-Met and Glu141-to-Lys substitutions affect substrate binding and ATP interaction. Although performing these experiments is currently beyond our technical capabilities, future collaborative research with experts in biochemistry, transport kinetics, and structural biology is anticipated to play an important role in fully elucidating the underlying mechanisms.

## 4. Materials and Methods

### 4.1. Preparation of pcDNA5/FRT Containing ABCG2 (12M/141K) Variant cDNA

The expression vector pcDNA5/FRT/*ABCG2 (12M/141K)* was generated from a previously constructed pcDNA5/FRT/*ABCG2 (V12M)* plasmid, as described previously [12,36]. Based on the reference sequence information for the Q141K variant of the ABCG2 gene available in the NCBI dbSNP database, site-directed mutagenesis was performed to introduce this nonsynonymous single-nucleotide polymorphism (SNP) using PrimeSTAR^®^ Max DNA Polymerase (Takara Bio Inc., Otsu, Japan) and mutation-specific primers. Following PCR amplification under the optimized conditions, the reaction mixture was treated with DpnI endonuclease to selectively digest the methylated parental plasmid DNA (pcDNA5/FRT/*ABCG2 [V12M]*). The presence of the desired mutations and integrity of the resulting constructs were confirmed by DNA sequencing using Applied Biosystems 3130 and 3130 xl Genetic Analyzers (Applied Biosystems, Foster City, CA, USA).

### 4.2. Cell Culture

Flp-In™-293 cells (Invitrogen, Thermo Fisher Scientific, Waltham, MA, USA) were cultured in high-glucose Dulbecco’s Modified Eagle’s Medium (DMEM) supplemented with 10% heat-inactivated fetal bovine serum (FBS), 4 mM L-glutamine, 100 U/mL penicillin, 100 μg/mL streptomycin, 250 ng/mL amphotericin B, and 100 μg/mL zeocin, in a humidified atmosphere containing 5% CO_2_. To maintain Flp-In-293/ABCG2 (12M/141K) cells, 50 μg/mL hygromycin B was used instead of zeocin. Cell viability was assessed by trypan blue exclusion assay using a hemocytometer, and only viable cells were used in subsequent experiments. Zeocin was obtained from Invitrogen (Thermo Fisher Scientific, Waltham, MA, USA). The Antibiotic-Antimycotic Mixed Stock Solution (100×), containing 10,000 U/mL penicillin, 10,000 μg/mL streptomycin, and 25,000 ng/mL amphotericin B, L-glutamine, and high-glucose DMEM were purchased from Nacalai Tesque, Inc. (Kyoto, Japan). Fetal bovine serum (FBS) was obtained from Equitech-Bio, Inc. (Kerrville, TX, USA).

### 4.3. Generation of Cells Expressing ABCG2 (12M/141K) Variant

Flp-In™-293 cells were seeded into 35 mm culture dishes (TrueLine, Baton Rouge, LA, USA) at a density of 1 × 10^6^ cells per dish and pre-incubated for 24 h. Cells were subsequently co-transfected with the pcDNA5/FRT/*ABCG2 (12M/141K)* expression vector and the Flp recombinase expression plasmid pOG44 using Lipofectamine™ 2000 (Invitrogen, Thermo Fisher Scientific, Waltham, MA, USA) in accordance with the manufacturer’s instructions. Following transfection, the cells were selected with 50 μg/mL hygromycin B, and the resulting hygromycin B-resistant colonies were collected, expanded, and designated as Flp-In-293/ABCG2 (12M/141K) cells for use in subsequent experiments.

Lipofectamine™ 2000 and pOG44 were purchased from Invitrogen (Thermo Fisher Scientific, Waltham, MA, USA). Hygromycin B was obtained from Nacalai Tesque, Inc. (Kyoto, Japan).

### 4.4. Total RNA Preparation and First-Strand cDNA Synthesis

Flp-In-293/ABCG2 (12V/141Q) (WT), Flp-In-293/ABCG2 (12M/141Q), Flp-In-293/ABCG2 (12V/141K), and Flp-In-293/ABCG2 (12M/141K) cells were seeded into 35 mm culture dishes (TrueLine, Baton Rouge, LA, USA) at a density of 1 × 10^6^ cells per dish and pre-incubated for 3 days. The cells were then harvested together with the culture medium into 1.5 mL microcentrifuge tubes, centrifuged at 300× *g* for 5 min at 4 °C, and washed twice with 1 mL of phosphate-buffered saline (PBS) without calcium and magnesium [PBS (–)]. The resulting cell pellets were stored at −80 °C until total RNA extraction. Total RNA was isolated from the frozen cell pellets using 600 μL of lysis/binding buffer from the High Pure RNA Isolation Kit (Roche Diagnostics, Mannheim, Germany) according to the manufacturer’s protocol. RNA concentrations were quantified using a DU640 spectrophotometer (Beckman Coulter, Fullerton, CA, USA). The extracted total RNA was subsequently used for first-strand complementary DNA (cDNA) synthesis using the High-Capacity cDNA Reverse Transcription Kit (Thermo Fisher Scientific Inc., Waltham, MA, USA), according to the manufacturer’s instructions.

### 4.5. Quantitative Evaluation of ABCG2 mRNA

The expression levels of *ABCG2* mRNA were quantified using the 7500 Fast Real-Time PCR System (Applied Biosystems, Foster City, CA, USA). Reactions were performed with the GoTaq^®^ qPCR Master Mix, 2× (Promega, Tokyo, Japan), which employs the SYBR Green detection method, and specific primers targeting either ABCG2 or GAPDH.

The GoTaq^®^ qPCR Master Mix, 2× was obtained from Promega (Tokyo, Japan). Primer sets for ABCG2 (Forward primer, 5′-GGAGGCCTTGGGATACTTTGA; Reverse primer, 5′-TCTATGAGTGGCTTATCCTGCTTG) and GAPDH (Forward primer, 5′-GCACCGTCAAGGCTGAGAAC; Reverse primer, 5′-TGGTGAAGACGCCAGTGGA) were purchased from Takara Bio Inc. (Otsu, Japan).

### 4.6. MTT Assay

Flp-In-293/ABCG2 (12V/141Q) (WT), Flp-In-293/ABCG2 (12M/141Q), Flp-In-293/ABCG2 (12V/141K), and Flp-In-293/ABCG2 (12M/141K) cells were seeded into 96-well plates (Thermo Fisher Scientific, Waltham, MA, USA) at a density of 5 × 10^5^ cells/well, cultured for 24 h, and subsequently treated with various concentrations of anticancer drugs (mitoxantrone and SN-38) for 72 h. The final drug concentrations ranged from 0 M (control) to 1000 nM for mitoxantrone and 100 nM for SN-38.

Following drug treatment, the cells were incubated with 500 μg/mL MTT for 3 h and then lysed with 10% sodium dodecyl sulfate (SDS). The plates were then incubated overnight at 37 °C in a humidified atmosphere containing 5% CO_2_. The absorbance at 570 nm was measured using a Multiskan Jax spectrophotometer (Thermo Fisher Scientific) with a reference wavelength of 630 nm to quantify the amount of formazan generated from MTT metabolism in each well. Cell viability was calculated as a percentage of the control group based on the absorbance at 570 nm (reference: 630 nm). The cytotoxicity of mitoxantrone and SN-38 was evaluated by determining the half-maximal effective concentration (EC_50_), defined as the drug concentration required to reduce cell viability by 50% based on the survival curve.

Mitoxantrone and SN-38 were purchased from Wako Pure Chemical Industries, Ltd. (Osaka, Japan) and kindly provided by Yakult Honsha Co. (Tokyo, Japan), respectively. 3-[4,5-dimethylthiazol-2-yl]-2,5-diphenyltetrazolium bromide (MTT) was obtained from Sigma-Aldrich Co. (St. Louis, MO, USA).

### 4.7. Cell Lysate Preparation for SDS-PAGE

Flp-In-293/ABCG2 (12V/141Q) (WT), Flp-In-293/ABCG2 (12M/141Q), Flp-In-293/ABCG2 (12V/141K), and Flp-In-293/ABCG2 (12M/141K) cells were seeded into 35 mm culture dishes (TrueLine, Baton Rouge, LA, USA) at a density of 1 × 10^6^ cells per dish and pre-incubated for 3 days. Cells were harvested together with the culture medium in 1.5 mL microcentrifuge tubes, centrifuged at 300× *g* for 5 min at 4 °C, and washed twice with 1 mL of PBS (–) (phosphate-buffered saline without calcium and magnesium). The resulting cell pellets were resuspended and lysed in buffer containing 50 mM Tris-HCl (pH 7.6), 5 mM EDTA (pH 8.0), 120 mM NaCl, 1% Triton X-100, 1 mM DTT, and commercially available protease and phosphatase inhibitor cocktails.

Cell lysis was facilitated by homogenization using a 27-gauge needle (10 passes). The lysates were centrifuged at 3000 rpm for 10 min at 4 °C, and the supernatants were collected as total cell lysates. Protein concentrations were determined by Bradford assay using bovine serum albumin (BSA) as a standard. To evaluate the expression level of ABCG2, 50 μg of total protein was treated with PNGase F at 37 °C for 10 min to remove N-linked glycan moieties. The samples were then mixed with SDS-PAGE loading buffer containing 10% (*v*/*v*) 2-mercaptoethanol (Daiichi Pure Chemicals Co., Ltd., Tokyo, Japan) and stored at −30 °C until use in Western blot analysis.

### 4.8. Evaluation of Expression Status and Levels of ABCG2

Cell lysates were prepared in triplicate from each cell line, and 5 μg aliquots from the triplicates were pooled within each group to generate representative cell lysate mixtures. These mixtures were subjected to SDS–PAGE on a 7.5% polyacrylamide gel, followed by electrotransfer onto nitrocellulose membranes (GE Healthcare UK Ltd., Bucks, UK).

Western blotting was performed following membrane blocking, in which the membranes were incubated in TBST (50 mM Tris-HCl, 150 mM NaCl, and 0.05% [*v*/*v*] Tween 20) supplemented with 5% (*w*/*v*) skimmed milk powder for at least 1 h at room temperature, followed by overnight incubation at 4 °C. After washing with TBST, the membranes were incubated with 1:1000-diluted primary antibodies, either a monoclonal anti-ABCG2 antibody (BXP-21; ALEXIS Co., Lausen, Switzerland) or anti-GAPDH antibody (Clone 6C5, mouse monoclonal, IgG2b; American Research Products, Inc., Waltham, MA, USA), in 5% (*w*/*v*) skim milk-containing TBST with gentle agitation for 1 h at room temperature.

Following primary antibody incubation, the membranes were washed again with TBST and incubated with HRP-conjugated anti-mouse IgG secondary antibody (1:1000; Cell Signaling Technology, Inc., Danvers, MA, USA) under the same conditions. Immunoreactive bands were visualized using Western Lightning Chemiluminescent Reagent Plus (PerkinElmer Life and Analytical Sciences, Boston, MA, USA) and detected using a WSE-6100 LuminoGraph I imaging system (Atto Corp., Tokyo, Japan). ImageJ software (version 1.54) (Wayne Rasband, NIH, Bethesda, MD, USA) was used for densitometric quantification of the signal intensities corresponding to ABCG2 and GAPDH.

### 4.9. Statistical Analysis

Statistical analyses were performed using JSTAT software (version 20.0) developed by Masato Sato. One-way analysis of variance (ANOVA) followed by Tukey’s honestly significant difference (HSD) test was used to assess the group differences. A *p*-value less than 0.05 was considered statistically significant in all analyses.

## 5. Conclusions

In this study, we established Flp-In™-293 cell lines expressing ABCG2 (12M/141K) and compared their expression and functional properties with those of cells expressing ABCG2 (12V/141Q) (WT), ABCG2 (12M/141Q), and ABCG2 (12V/141K). We demonstrated that ABCG2 (12M/141K) exhibited expression levels comparable to those of the other variants at both mRNA and protein levels. ABCG2 (12M/141K) conferred significantly higher resistance to mitoxantrone and SN-38 compared to Flp-In-293/Mock cells, indicating that ABCG2 (12M/141K) functions actively in these cells. Importantly, while the overall drug resistance profile of ABCG2 (12M/141K) was similar to that of ABCG2 (12V/141Q) (WT), resistance was lower than that of ABCG2 (12M/141Q) and showed a trend toward higher mitoxantrone resistance compared to ABCG2 (12V/141Q) (WT) (*p* = 0.066), suggesting a possible specific effect of the *ABCG2 (12M/141K)* haplotype. These results provide new insights into the combined functional impact of V12M and Q141K polymorphisms on ABCG2-mediated drug resistance.

The function of ABCG2 (12M/141K) and the drug resistance profiles of cells expressing this haplotype remain unclear. rs2231137 (V12M) and rs2231142 (Q141K) are two common and functionally important variants associated with disease susceptibility and drug response across diverse pathological contexts, including gout [38,40], cancer [27,28,29,30,31,32,33,34], chronic kidney disease [41], idiopathic male infertility [42], preeclampsia [43], and type 2 diabetes [44]. This study enhances our understanding of the potential role of nonsynonymous SNP combinations in modulating ABCG2 function and drug resistance. Moreover, these findings may inform the development of personalized chemotherapy strategies, such as selecting optimal anticancer agents or adjusting dosages based on *ABCG2* haplotype status, and contribute to novel approaches to overcome ABCG2-mediated drug resistance in cancer treatments.

## Figures and Tables

**Table 1:** Anticancer drug resistance profiles (EC_50_ values) of Flp-In™-293 cells expressing ABCG2 variants.

Cell Type	EC_50_ (nM)					
	Mitoxantrone			SN-38		
Mock	4.67	±	1.27	1.97	±	0.105
ABCG2 (12V/141Q)(WT)	32.9	±	7.75 *	8.71	±	0.613 *
ABCG2 (12M/141Q)	91.3	±	14.2 *^,^ **	25.8	±	3.63 *^,^ **
ABCG2 (12V/141K)	43.2	±	6.07 *	9.20	±	0.572 *
ABCG2 (12M/141K)	49.8	±	7.27 *	11.8	±	1.78 *

SN-38, 7-ethyl-10-hydroxy-camptothecin. The drug resistance profiles of cells established using the Flp-In™ system were evaluated by the MTT assay. Data are expressed as mean ± S.D. (*n* = 5). Statistical analyses were performed using one-way ANOVA followed by Tukey’s HSD test. * *p* < 0.01 vs. Mock group; ** *p* < 0.01 vs. wild-type (WT).

## Data Availability

The raw data supporting the conclusions of this article will be made available by the authors on request.

## Abbreviations

The following abbreviations are used in this manuscript:

ABC	ATP-binding cassette
ANOVA	Analysis of variance
BCRP	Breast cancer resistance protein
BSA	Bovine Serum Albumin
DMEM	Dulbecco’s modified Eagle’s medium
FRT	Flp recombination target
GAPDH	glyceraldehyde-3-phosphate dehydrogenase
HRP	horseradish peroxidase
HSD	Honestly Significant Difference
MTT	3-[4,5-dimethylthiazol-2-yl]-2,5-diphenyltetrazolium bromide
MXR	Mitoxantrone resistance-associated protein
PBS(–)	phosphate-buffered saline without calcium and magnesium
qPCR	quantitative real-time polymerase chain reaction
SDS	Sodium dodecyl sulfate
SDS-PAGE	Sodium Dodecyl Sulfate -Polyacrylamide Gel Electrophoresis
TBS	Tris-buffered saline
TBST	TBS with 0.05% (*v*/*v*) Tween 20
WT	Wild-Type
